# Human Diseased Articular Cartilage Contains a Mesenchymal Stem Cell-Like Population of Chondroprogenitors with Strong Immunomodulatory Responses

**DOI:** 10.3390/jcm8040423

**Published:** 2019-03-28

**Authors:** Paola De Luca, Dimitrios Kouroupis, Marco Viganò, Carlotta Perucca-Orfei, Lee Kaplan, Luigi Zagra, Laura de Girolamo, Diego Correa, Alessandra Colombini

**Affiliations:** 1 IRCCS Istituto Ortopedico Galeazzi, Orthopaedic Biotechnology Lab, Milan 20161, Italy; deluca.paola@grupposandonato.it (P.D.L.); marco.vigano@grupposandonato.it (M.V.); carlotta.perucca@grupposandonato.it (C.P.-O.); alessandra.colombini@grupposandonato.it (A.C.); 2 Department of Orthopedics, UHealth Sports Medicine Institute, University of Miami, Miller School of Medicine, Miami, FL 33146, USA; dxk504@med.miami.edu (D.K.); kaplan@med.miami.edu (L.K.); 3 Diabetes Research Institute & Cell Transplant Center, University of Miami, Miller School of Medicine, Miami, FL 33136, USA; 4 IRCCS Istituto Ortopedico Galeazzi, Hip Department, Milan 20161, Italy; luigi.zagra@fastwebnet.it

**Keywords:** cartilage cells, cartilage-derived stem/progenitor cells, mesenchymal stem cells, stemness, inflammation, secretome, immunomodulation

## Abstract

Background: osteoarthritic human articular cartilage (AC)-derived cartilage cells (CCs) with same-donor bone marrow (BMSCs) and adipose tissue (ASCs)-derived mesenchymal stem cells were compared, in terms of stemness features, and secretory and immunomodulatory responses to inflammation. Methods: proteoglycan 4 (PRG4) presence was evaluated in AC and CCs. MSCs and CCs (*n* = 8) were cultured (P1 to P4) and characterized for clonogenicity, nanog homeobox (*NANOG*), and POU class 5 homeobox 1 (*POU5F1*) expression, immunotypification, and tri-lineage differentiation. Their basal and interleukin-1β (IL-1β)-stimulated expression of matrix metalloproteases (MMPs), tissue inhibitors (TIMPs), release of growth factors, and cytokines were analyzed, along with the immunomodulatory ability of CCs. Results: PRG4 was mainly expressed in the intact AC surface, whereas shifted to the intermediate zone in damaged cartilage and increased its expression in CCs upon culture. All cells exhibited a similar phenotype and stemness maintenance over passages. CCs showed highest chondrogenic ability, no adipogenic potential, a superior basal secretion of growth factors and cytokines, the latter further increased after inflammatory stimulation, and an immunomodulatory behavior. All stimulated cells shared an increased MMP expression without a corresponding TIMP production. Conclusion: based on the observed features, CCs obtained from pathological joints may constitute a potential tissue-specific therapeutic target or agent to improve damaged cartilage healing, especially damage caused by inflammatory/immune mediated conditions.

## 1. Introduction

The study of resident adult stem cells and progenitors in tissues with limited self-renewal ability, such as articular cartilage (AC), constitutes a partially explored research field with constant growing interest [1]. Those specific cells can become a target or be part of regenerative-based novel treatments for AC damage. AC is a highly specialized tissue with a healing capacity after injury present during embryonic development [2], reduced after birth, and virtually lost thereafter [3]. AC contains a mixed population of cartilage cells (CCs) composed by differentiated matrix-embedded chondrocytes and a pool of resident cartilage-derived stem/progenitor cells (CSPCs). The latter cell population presents a stem-like immuno-phenotype, with chondrogenic potential and the ability to respond to injury with an increased migratory behavior. CSPCs were first identified by observing that while chondrocytes in culture were rapidly losing their phenotype (i.e., dedifferentiation), they showed colony-forming cells with multi-differentiation potential [4]. Furthermore, chondroprogenitors were found at the healthy AC surface [5], associated with the expression of proteoglycan 4 (*PRG4*) encoding for proteoglycan 4 or lubricin [6] also produced by migratory chondrogenic progenitors in damaged AC [7].

These cells found within normal and osteoarthritic tissues are CD105^+^ and CD166^+^, show a differentiation capacity comparable to BMSCs, and participate in reparative attempts to restore structural integrity and tissue homeostasis [4,7,8,9,10,11]. In various clinical scenarios, CCs are processed and used therapeutically, such as in autologous chondrocyte implantation (ACI), in which harvested and culture-expanded chondrocytes are then used to treat AC focal defects and even osteoarthritis (OA) with varying success rates [12,13].

The use of mesenchymal stem cells (MSCs) from different sources has been proposed for years as a viable therapeutic alternative to treat AC damage, however, various challenges are still present [14]. MSCs can be used therapeutically given their in vitro chondrogenic potential and established immunomodulatory and trophic properties (collectively called “medicinal”) [15,16]. The medicinal properties of MSCs are tightly associated with a strategic perivascular localization where they exhibit a pericytic phenotype [17], as well as an environmental sensing capacity that allows them to modulate local responses to tissue disruption (e.g., inflammation and trauma) [15]. In particular, MSCs are molecularly equipped to recognize, home, and engraft to distant injured tissues, where they exert similar activities after retaking the key perivascular localization [18,19]. MSCs’ medicinal activities help to maintain or re-establish tissue homeostasis through the paracrine secretion of bioactive molecules that modulate local immune responses, stimulate angiogenesis, and promote tissue-specific progenitor proliferation, while inhibiting cell apoptosis and tissue fibrosis. MSCs are therefore able to induce the establishment of a “regenerative microenvironment” [16,20].

The avascular nature of AC limits the extrapolation of the concept of a responsive perivascular MSC to local inflammation/tissue imbalance during AC damage. Nevertheless, as stated above, resident populations of mitotic and plastic CSPCs have shown to be present within the damaged tissue, apparently migrating from surrounding “non-weight bearing healthy” tissue and/or from gaps at the tidemark [7,10,11]. CSPCs represent an intermediate cell population between differentiated chondrocytes and MSCs, which can be either ex vivo processed or in vivo stimulated to induce tissue healing and homeostasis at different stages of AC damage. Despite reported similarities of CSPCs with MSCs, a full characterization of CCs as a mixed cell-based product, including their secretory response to an inflammatory environment and subsequent medicinal activities, have not been thoroughly investigated. 

Consequently, we aimed to compare side-by-side human-derived CCs (from healthy AC surrounding advanced osteoarthritic tissue) with same-donor BMSCs and MSCs derived from adipose tissue (ASCs), with regards to stemness features in culture, phenotypic display, multi-differentiation potential, and extracellular matrix (ECM) remodeling of protein gene expression. Due to the emerging role of the MSC-derived secretome as a promising immunomodulatory/trophic cell-free approach for OA treatment [21], we further assessed their comparative ability to sense and respond to an in vitro inflammatory environment, by interrogating the composition of the conditioned media produced by the three cell types, under the hypothesis that CCs could not only harbor a progenitor cell, but also exert immunomodulatory effects as MSCs do.

## 2. Materials and Methods

### 2.1. Histological Analysis

Formalin-fixed, ethylene diamine tetraacetic acid (EDTA)-decalcified (6 weeks), paraffin-embedded, 4 µm-sectioned explanted femoral heads from 3 patients (2 males and 1 female, age range 44–74 years) were stained with hematoxylin and eosin and immunohistochemically for type II collagen and PRG4 (lubricin) assessment. Briefly, sections were blocked (2% bovine serum albumin (BSA), incubated with a rabbit polyclonal anti-type II collagen (AB34712, Abcam, Cambridge, UK) and anti-lubricin primary antibody (AB28484, Abcam, Cambridge, UK) diluted in 5% phosphate-buffered saline (PBS)-BSA (1:100 and 1:500, respectively) for 1 h at room temperature, followed by washing with PBS buffer (PBS 1 × + Tween20) and incubation for 30 min with a biotinylated goat anti-rabbit IgG secondary antibody (1:200 diluted in PBS 2% BSA, INVC-BA-1000-MM15, Vector Laboratories, Burlingame, CA, USA). Diaminobenzidine (ImmPACT DAB peroxidase, Vector Labs, Burlingame, CA, USA) was used as a chromogenic substrate of the peroxidase reaction, and all sections were counterstained with Mayer’s hematoxylin, dehydrated, and mounted.

### 2.2. Cell Isolation and Expansion

The study was approved by the local Institutional Review Board (M-SPER-015, for use of discarded biological material), involving 8 consented/de-identified patients (5 females and 3 males, age range 41–74 years), with OA (Kellgren Lawrence III or IV) undergoing total hip arthroplasty. AC was harvested with a scalpel from non-weight bearing superficial areas of femoral head/neck, excluding the subchondral bone; bone marrow from femoral channel after neck resection; and subcutaneous adipose tissue from local hip fat deposit. CCs were isolated by enzymatic digestion of harvested AC (37 °C, 22 h) with 0.15% w/v type II collagenase (Worthington Biochemical, Lakewood, NJ, USA) [22], then cultured in control medium consisting of 4.5 mg/mL high Glucose DMEM supplemented with 10% FBS (Lonza), 0.29 mg/mL L-glutamine, 100 U/mL penicillin, 100 µg/mL streptomycin, 10 mM 4-(2-hydroxyethyl)piperazine-1-ethanesulfonic acid (HEPES), 1 mM sodium pyruvate (all reagents from Life Technologies, Carlsbad, CA, USA). Bone marrow was washed in PBS, centrifuged, and BMSCs selected for plastic adherence [23]. ASCs were isolated by enzymatic digestion of harvested adipose tissue (37 °C, 30 min) using 0.075% w/v type I collagenase (Worthington Biochemical, Lakewood, NJ, USA) as previously reported [24,25]. ASCs and BMSCs were then cultured in minimum essential medium (αMEM) supplemented as described above, adding 5 ng/mL fibroblast growth factor 2 (FGF-2) (PeproTech, Rocky Hill, NJ, USA) to preserve the chondrogenic potential [26,27]. All cell types were expanded up to four passages (indicated as P1–P4).

### 2.3. Clonogenic Ability

A colony-forming unit-fibroblast (CFU-F) assay was performed [24] at P1 and P3. The cells were plated at different low densities (range, 48–12 cells/cm^2^) and cultured in control medium with 20% FBS. After 14 days, the cells were fixed with 10% neutral buffered formalin and stained with Gram’s crystal violet (Sigma-Aldrich, Saint Louis, MO, USA). CFU-F frequency was established by counting the colonies and expressing them as a percentage relative to the number of seeded cells.

### 2.4. Immunophenotype

Flow cytometry analysis was conducted at P4 on 2.5 × 10^5^ cells incubated with anti-human primary monoclonal antibodies: CD14-FITC, CD34-biotinylated, CD44-FITC, CD45-FITC, CD71-biotinylated, CD105-biotinylated, CD166-FITC (Ancell Corporation, Bayport, MN, USA), CD90-FITC and CD73-PE (Miltenyi Biotec, Bergisch Gladbach, Germany), and CD151 (R&D Systems, Minneapolis, MN, USA). Biotinylated stained cells were incubated with streptavidin-PE (Ancell Corporation, Bayport, MN, USA), whereas anti-CD151 stained samples were incubated with a FITC-conjugated goat anti-mouse secondary antibody (Ancell Corporation, Bayport, MN, USA). Background fluorescence was established by unstained cells as negative controls and data acquired using a FACSCalibur flow cytometer (BD Biosciences, San Jose, CA, USA) collecting a minimum of 10,000 events. The analysis was performed using CellQuest software (BD Biosciences, San Jose, CA, USA).

### 2.5. Multi-Lineage Differentiation

At P1 and P3, 3 × 10^3^ cells/cm^2^ were plated and differentiated for 14 days either in adipogenic or osteogenic medium as previously reported [28]. A commercially available medium (Miltenyi Biotec, Bergisch Gladbach, Germany) optimized for the generation of adipocytes from human MSCs was used as an adipogenic differentiation control (*n* = 3 at P1). Lipid vacuoles were quantified by Oil Red O staining and calcified matrix deposition was measured using Alizarin Red-S staining and absorbances were read at 490 nm and at 570 nm, respectively (Perkin Elmer Victor X3 microplate reader). 

Pellet cultures at P1 and P3 were obtained by centrifugation of 4 × 10^5^ cells, maintained for 28 days in chondrogenic medium, following an already published protocol [29]. An additional 10 ng/mL of bone morphogenetic protein 6 (BMP-6) (PeproTech, Rocky Hill, NJ, USA) was added to the ASCs [30].

To evaluate the glycosaminoglycans (GAGs) deposition, pellets were fixed, embedded in paraffin, sectioned at 4 µm, and stained with Alcian Blue (Sigma-Aldrich, Saint Louis, MO, USA). For GAGs’ quantification, pellets were digested (16 h, 60 °C) in PBE buffer containing L-cysteine (Sigma-Aldrich, Saint Louis, MO, USA) and papain (Worthington Biochemical Co., Lakewood, NJ, USA). Samples were incubated with dimethylmethylene blue (Sigma-Aldrich, Saint Louis, MO, USA) and absorbance was read at 500 nm.

### 2.6. In Vitro Model of Inflammation

Cells at P3 were stimulated with 1 ng/mL of IL-1β for 48 h [31,32], after which both supernatant and cells were collected.

### 2.7. Gene Expression Analysis

Total RNA was isolated from cell lysates using the PureLink® RNA Mini Kit (Life Technologies, Carlsbad, CA, USA) and quantified spectrophotometrically (NanoDrop, Thermo Scientific, Waltham, MA, USA). RNA was reverse-transcribed to cDNA employing the iScript cDNA Synthesis Kit (Bio-Rad Laboratories, Hercules, CA, USA). Gene expression was evaluated by real time PCR (StepOne Plus, Life Technologies, Carlsbad, CA, USA), with cDNA incubated with a PCR mixture, including TaqMan® Gene Expression Master Mix and TaqMan® Gene Expression Assays (Life Technologies, Carlsbad, CA, USA). 

Expression levels of *NANOG*, Hs04260366_g1, *POU5F1*, Hs04260367_gh in all cells, and of *PRG4*, Hs00981633_m1 in CCs at P1 and P3 were evaluated. *MMP1*, Hs00899658_m1, *MMP3*, Hs00968305_m1, *MMP13*, Hs00233992_m1, *TIMP1*, Hs00171558_m1, *TIMP3*, and Hs00165949_m1 were analyzed at P3 with or without IL-1β stimulation in all cell types. *TBP*, Hs00427620_m1 was selected as a housekeeping gene [33]. Data were expressed according to the dCt or ddCt (*PRG4*) method.

### 2.8. Protein Array

Commercially available multiplex ELISA-based protein arrays for growth factors (GFs) (Table S1) and for inflammation mediators (Table S2) (RayBio^®^ C-Series, RayBiotech, Norcross, GA, USA) were used to evaluate basal and post-IL-1β stimulation relative levels in media obtained from all cells (4 donors run in 3 technical replicates pooled) following the manufacturer’s instructions, and normalized by the total protein content assessed through bicinchoninic assay (BCA). Samples were exposed to a FluorChem E chemiluminescence imaging system (ProteinSimple, San Jose, CA, USA) to quantify the mean spot pixel signal density using the protein array analyzer for ImageJ (ImageJ, NIH website). The signal intensity for each protein spot is proportional to the relative concentration of the antigen in the sample. 

### 2.9. Determination of IL-1Ra

The levels of soluble interleukin 1 receptor antagonist (IL-1Ra) (detection range of 23–1500 pg/mL) in cell culture medium treated or not treated with IL-1β for 48 h were determined by commercially available enzyme-linked immunosorbent assays (ELISAs) according to the manufacturer’s instructions (PeproTech, Rocky Hill, NJ, USA).

### 2.10. Immunopotency Assay (IPA)

CCs (7 cell populations) were seeded in 96-well plates at a density of 2 × 10^4^ or 5 × 10^4^ in 100 µL control medium, and 24 h later stimulated with 1 ng/mL of IL-1β. After 48 h, medium containing IL-1β was removed and 1 × 10^5^ peripheral blood lymphocytes (PBLs) pre-treated with 0.1 µg/mL anti-human CD3/CD28 antibodies (Mabtech AB) were added to CCs cultures. After 72 h, PBLs’ proliferation was evaluated by analyzing BrdU incorporation with the Cell Proliferation ELISA, BrdU Kit (Roche) following the manufacturer’s protocol. The absorbance was measured at 370 nm (Perkin Elmer Victor X3 microplate reader). The experiments were replicated with PBLs derived from 2 different donors. Stimulated PBLs cultured in the absence of CCs were used as positive controls, while non-stimulated samples (cultured in the absence of anti-CD3/CD28) were used as negative controls.

### 2.11. Statistical Analysis

Data are expressed as mean ± standard deviation (SD). Normal distribution of values was assessed by the Kolmogorov-Smirnov normality test. Statistical analysis was performed using paired and unpaired Student’s *t*-test for normally distributed data and Wilcoxon (for paired data) or Mann Whitney (for unpaired data) test in the absence of a normal distribution; one-way ANOVA was used for multiple comparisons. Pearson’s (for paired data) or Spearman’s (for unpaired data) correlation of *PRG4* expression and inflammatory biomarkers was performed (GraphPad Prism v5.00, San Diego, CA, USA). Level of significance was set at *p* < 0.05 (* *p* < 0.05, ** *p* < 0.01, *** *p* < 0.001). The number of data used for the statistical analyses is indicated in the figure legends and corresponds to independent experiments [34].

## 3. Results

### 3.1. PRG4 (lubricin) Expression Shifts from Healthy to Damaged AC and Increases in CCs during in vitro Culture

**Figure 1:**
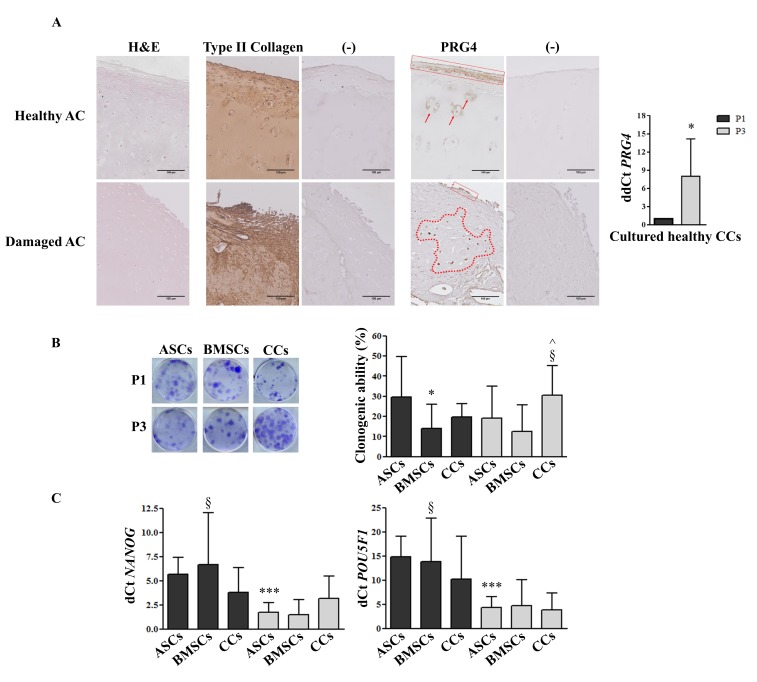
PRG4 expression, clonogenic ability, and stemness marker expression. (**A**) Representative immunohistological distribution of type II collagen and PRG4 in healthy and damaged AC (scale bars correspond to 100 µm), and PRG4 expression in culture-expanded CCs (*n* = 4). (−) indicates negative control (secondary antibody only). (**B**) Clonogenic ability and (**C**) stemness marker expression of adipose (ASCs), bone marrow (BMSCs)-derived MSCs and cartilage cells (CCs) obtained from the same eight donors. Cells were analyzed at passage 1 (P1) and passage 3 (P3). * *p* < 0.05, *** *p* < 0.001 vs. ASCs at P1, § *p* < 0.05 vs. BMSCs at P3, ^ *p* < 0.05 vs. CCs at P1. Data are represented as mean ± SD (*n* = 8).

The intact portion of cartilage (non-weight bearing area) was characterized by normal cartilage tissue morphology rich in type II collagen, with the highest PRG4 presence in the upper zone and mildly in the intermediate zone in some cells. In the interface portion, between intact and damaged cartilage, the tangential layer was missing and the tidemark in the pathological side was not distinguishable, with PRG4 localized in a thinner superficial area compared with intact AC (data not shown). In the damaged AC sections, the tissue structure appeared non-homogeneous, exemplified by a distorted superficial zone, with PRG4 expression randomly distributed in the intermediate zone within CCs (Figure 1A). Notably, the *PRG4* expression level was positive in CCs after isolation (control) and exhibited a significant (*p* < 0.05) upregulation (8-fold) after three culture passages (Figure 1A). With the exception of IL-4 (Pearson’s *r* = −0.98, *n* = 4 donors), no significant correlation between the inflammatory biomarkers analyzed and the *PGR4* expression in expanded chondrocytes was observed.

### 3.2. CCs Formed Colonies, Expressed Stemness Markers, and Differentiated into Osteo- and Chondrogenic Lineage 

From P1 to P3, CCs showed a significant increase (*p* < 0.05) in clonogenic ability, with a significantly higher (*p* < 0.05) number of colonies in comparison with BMSCs at P3, while at P1, the number of ASC colonies was significantly higher (*p* < 0.05) in comparison with BMSCs (Figure 1B). Stemness markers, *NANOG* and *POU5F1 (Oct-4)*, showed a significant reduction with passages in ASCs (*p* < 0.001) and BMSCs (*p* < 0.05), but not in CCs. A trend of lower *NANOG* expression was observed at P1 in CCs in comparison with both BMSCs and ASCs, reverted later at P3, where CCs only maintained this expression (Figure 1C). 

**Figure 2:**
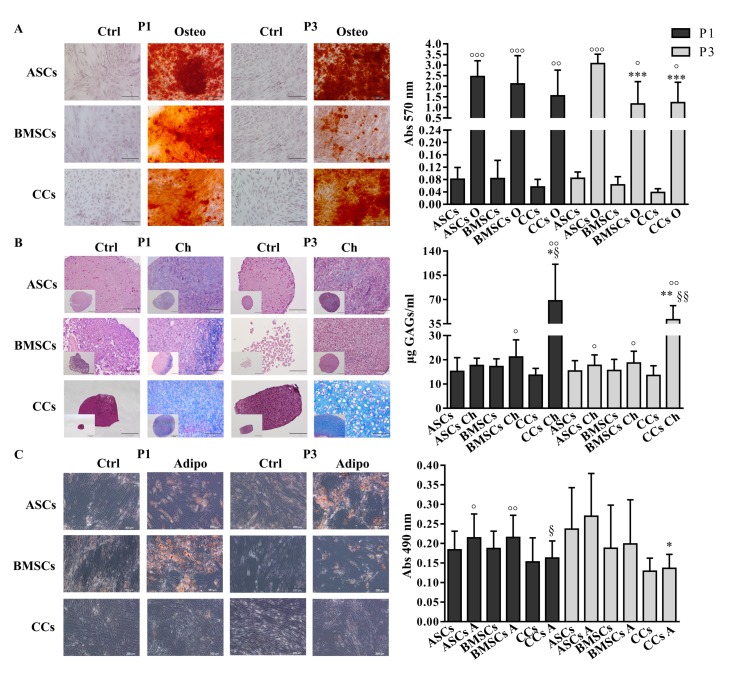
Multi-differentiation potential. (**A**) Osteogenic-O (Alizarin red staining), (**B**) Chondrogenic-Ch (Alcian blue staining and GAG quantification), and (**C**) Adipogenic-A (Oil O red staining) differentiation potential assessment. Adipose (ASCs)-, bone marrow (BMSCs)-derived MSCs and cartilage cells (CCs) obtained from the same eight donors were subjected to differentiation at P1 and P3. Scale bars correspond to 200 μm. Only scale bars of the biggest Chondrogenic-Ch images correspond to 100 µm. * *p* < 0.05, ** *p* < 0.01, *** *p* < 0.001 vs. ASCs, § *p* < 0.05, §§ *p* < 0.01 vs. BMSCs, ° *p* < 0.05, °° *p* < 0.01, °°° *p* < 0.001 vs. control. Data are represented as mean ± SD (*n* = 8).

All three cell types were able to differentiate into the osteogenic lineage at both P1 and P3, as demonstrated by the significant increase of calcified matrix deposition compared to untreated controls (ASCs P1 and P3 *p* < 0.001, BMSCs P1 *p* < 0.001 and P3 *p* < 0.05, CCs P1 *p* < 0.01 and P3 *p* < 0.05). In particular, ASCs showed a significantly higher amount (*p* < 0.001) of calcium deposition in comparison with CCs and BMSCs at P3 (Figure 2A). At P1, with the exception of ASCs, and at P3, all the cells differentiated toward the chondrogenic lineage, as shown by the significant increase of GAGs’ deposition compared to untreated controls (ASCs P3 *p* < 0.05, BMSCs P1 and P3 *p* < 0.05, CCs P1 and P3 *p* < 0.01). As expected, CCs showed significantly higher levels (*p* < 0.05 at P1, *p* < 0.01 at P3) of GAGs in comparison with both MSCs types (Figure 2B). Only ASCs and BMSCs at P1 showed appreciable and comparable signs of adipogenic differentiation compared with control cells (*p* < 0.05, *p* < 0.01, respectively). Indeed, significantly lower levels of lipid vacuole deposition were observed in CCs in comparison with BMSCs at P1 (*p* < 0.05) and ASCs at P3 (*p* < 0.05) (Figure 2C). The adipogenic differentiation was also verified with a commercial differentiation medium. While ASCs and BMSCs showed comparable results when treated either with the adipogenic medium currently used in our laboratory or the commercial medium (absorbance at 490 nm 0.140 ± 0.03 and 0.150 ± 0.02 in control and cultured ASCs in both adipogenic media, respectively; 0.154 ± 0.03 and 0.167 ± 0.04 and 0.191 ± 0.03 for control, standard medium, and commercial medium BMSCs, respectively; all differences were not statistically significant), CCs showed a better differentiation ability (*p* < 0.05) when cultured in our laboratory adipogenic medium (0.227 ± 0.02 and 0.219 ± 0.03 and 0.179 ± 0.005 for control, standard medium, and commercial medium CCs).

### 3.3. CCs and MSCs Share a Similar Immunophenotype

**Figure 3:**
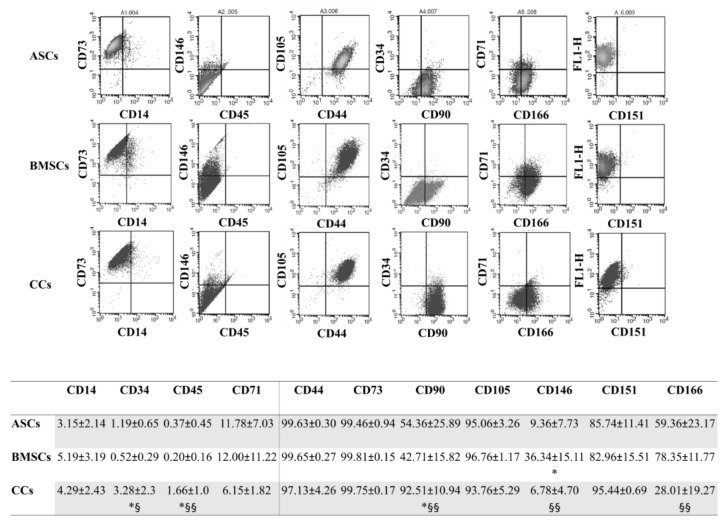
Immunophenotype. Representative expression of the typical pattern of MSC surface markers in all the analyzed cells, with a table reporting the percentages of positive cells for the whole panel of surface markers tested at P4. Adipose (ASCs)-, bone marrow (BMSCs)-derived MSCs, and cartilage cells (CCs). * *p* < 0.05 vs. ASCs, § *p* < 0.05, §§ *p* < 0.05 vs. BMSCs. Data are expressed as mean ± SD (*n* = 5).

At P4, as expected, both the MSC types were negative for CD14, CD34, CD45, and CD71 and positive for CD44, CD73, CD90 CD105, CD151, and CD166 expression. BMSCs were clearly positive for CD146, with significantly higher values in comparison to both ASCs (*p* < 0.05) and CCs (*p* < 0.01), which exhibited similar values. CCs showed a similar immunophenotypic pattern to MSCs. Notably, the expression of CD90 was significantly higher in CCs in comparison with both MSC types (*p* < 0.05 for ASCs and *p* < 0.01 for BMSCs), in which it was lower than expected. Finally, as already reported [8], CD105 and CD166 were positive in CCs, even with a significant lower (*p* < 0.01) expression of the latter compared with MSCs (Figure 3).

### 3.4. MSCs and CCs have Distinct Basal and IL-1β-Induced ECM Remodeling Predisposition

**Figure 4:**
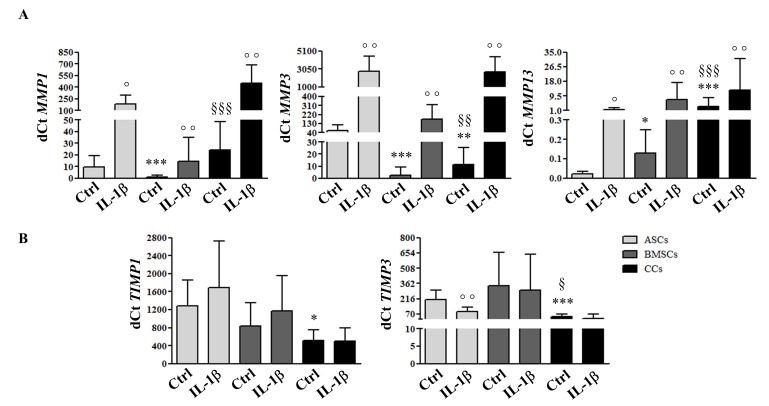
ECM remodeling molecular response to a pro-inflammatory stimulus. Expression of metalloproteases (*MMP1, MMP3, MMP13*) (**A**) and their inhibitors (*TIMP1, TIMP3*) (**B**) in adipose (ASCs)-, bone marrow (BMSCs)-derived MSCs, and cartilage cells (CCs) obtained from the same donors, assessed by quantitative real-time PCR at P3 before and after IL-1β stimulation. * *p* < 0.05, ** *p* < 0.01, *** *p* < 0.001 vs. ASCs, § *p* < 0.05, §§ *p* < 0.01, §§§ *p* < 0.001 vs. BMSCs, ° *p* < 0.05, °° *p* < 0.01 vs. control. Data are expressed as mean ± SD (*n* = 8).

In basal conditions, the highest and the lowest *MMP1* expression was demonstrated by CCs and BMSCs, respectively; for *MMP3*, the highest level was expressed by ASCs and the lowest by BMSCs. The highest and the lowest *MMP13* expression was shown by CCs and ASCs, respectively (Figure 4A). All the cells reacted to the inflammatory stimulation by upregulating *MMP1*, *3*, *13* expression in comparison with their basal levels. In the basal condition, the lowest expression of both *TIMP1* and *TIMP3* was observed in CCs. The highest expression was observed in ASCs and BMSCs. Interestingly, no change was observed for *TIMP1* and *TIMP3* levels after IL-1β treatment, with the exception of a significant downregulation of *TIMP3* in ASCs (Figure 4B).

### 3.5. Differential Production of Growth Factors (GF) in Basal and Pro-Inflammatory Conditions

**Figure 5:**
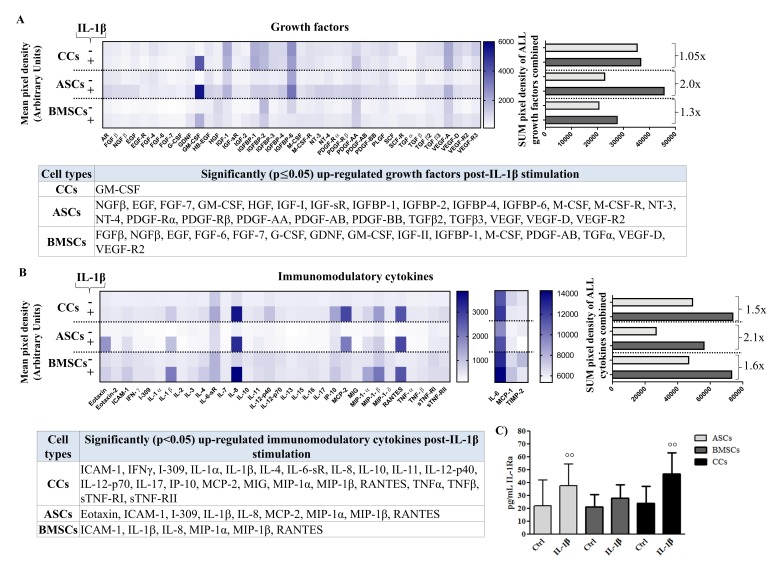
Secretome multiplex analysis. Secretion of growth factors of (**A**) inflammation-related cytokines (**B**) and IL-1Ra (**C**) in conditioned media obtained from adipose (ASCs)-, bone marrow (BMSCs)-derived MSCs, and cartilage cells (CCs) at basal (−) and post-stimulation with IL-1β (+). Growth factors and cytokines are presented as: overall heat maps of the mean pixel intensity, and arithmetic sum of the pixel intensity calculating the fold increase in the overall secretion from basal to post-stimulation; a table showing significantly upregulated molecules with stimulation (*n* = 4). For IL-1Ra, °° *p* < 0.01 vs. control, data are expressed as mean ± SD (*n* = 8).

In basal conditions, CCs showed a higher amount of total growth factors released compared to both two MSC types, based on the comparable total protein content assessed by BCA (Figure 5A and Figure S1 with statistical significance of all GFs among cell types). Lower levels of GFs belonging to the EGF, IGF, VEGF, and FGF families, as well as angiogenesis-related molecules (VEGF and VEGFRs), were observed in MSCs compared with CCs. Higher levels of GFs related to the promotion of cell proliferation, such as EGF, EGF R, FGF-4, FGF-6, FGF-7, and GDNF, were also observed in CCs when compared with BMSCs.

Post IL-1β stimulation, ASCs showed higher responsiveness compared to both BMSCs and CCs (Figure 5A), where the only growth factor increased after the inflammatory condition was granulocyte-macrophage colony-stimulating factor (GM-CSF). When comparing ASCs and BMSCs, 9 GFs showed a common upregulation, ASCs showed unique upregulation of 16 additional GFs, whereas BMSCs showed unique upregulation of 6 additional GFs. Interestingly, after IL-1β stimulation, angiogenic VEGF D, VEGF R2, and VEGF R3 increased in both MSC types whereas VEGF increased only in ASCs. Molecules belonging to the IGF and PDGF families were overall increased in both MSC types, but mainly in ASCs, whereas TGFβ-2 and TGFβ-3 increased only in ASCs (Figure 5A).

### 3.6. Differential Production of Cytokines in Basal and Pro-Inflammatory Conditions

In basal conditions, CCs showed similar levels to BMSCs and higher compared to ASCs (Figure 5B). Differences between pro- and anti-inflammatory cytokines were observed among cells (Figure S2 with statistical significance of all cytokines among cell types). A more general pro-inflammatory behavior in BMSCs was counteracted by higher levels of various immunomodulatory mediators, such as IL-10, sTNF-RI, sTNF-RI, I-309, Eotaxin, and others, some of them also highly present in CCs.

Post IL-1β treatment, all cell types were highly responsive in terms of changes in pro- and anti-inflammatory mediators (Figure 5B). Almost all of the immunomodulatory factors were increased in CCs, whereas only ICAM-1, IL-1b, IL-8, MIP-1-a, MIP-1-b, and RANTES increased in all three cell populations. Overall, BMSCs were the less responsive cells, with an increase of only six pro-inflammatory mediators, even in comparison with ASCs, which showed an increase in nine inflammatory mediators (Figure 5B, Figure S2). IL-1Ra, a critical anti-inflammatory cytokine, was upregulated after IL-1β treatment in all analyzed populations (increase vs controls of 1.7-fold for ASCs (*p* < 0.01), 1.3 fold for BMSCs and 2.0 fold for CCs (*p* < 0.01)) (Figure 5C).

### 3.7. CCs Stimulated with IL-1β Reduce the Proliferation of Activated PBLs in an IPA

**Figure 6:**
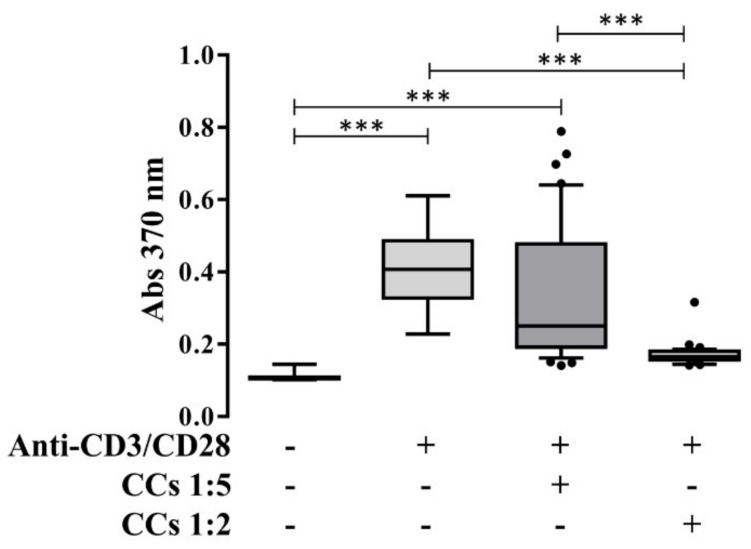
PBLs’ proliferation after interaction with CCs. Proliferation of PBLs in presence (+) or absence (−) of anti-CD3/CD28 and IL-1β-treated CCs in a ratio of 1:2 or 1:5 (PBLs:CCs). *** *p* < 0.001. Data are expressed as mean ± SD (*n* = 7).

Co-culturing CCs with PBLs at different ratios (immunopotency assay-IPA) demonstrated the ability of CCs stimulated with IL-1β to antagonize the proliferative response of activated PBLs by anti-CD3/CD28 antibodies. The higher reduction with respect to activated samples was obtained for the 1 CCs:2 PBLs condition, showing a dose-dependent effect (Figure 6).

## 4. Discussion

This study confirmed the presence of active chondrogenic progenitors within human cartilage obtained from OA patients, revealed their active and enhanced secretory responses to inflammation when compared with MSCs, and showed their immunomodulatory effects that may be harnessed under novel therapeutic schemes. In this view, CCs might not only be useful to derive novel and improve existing cell-based therapies for the treatment of cartilage defects, but also constitute a potential therapeutic target to enhance healing responses under various pathological circumstances, including inflammatory/immune mediated conditions.

The PRG4 shift in expression from the surface in intact/healthy AC, where it is generally associated with chondroprogenitor cells [6,35], to almost exclusively the intermediate zone in damaged AC highlights the migratory [7] and progenitor phenotype acquisition of those cells and their potential involvement in a remodeling/repair response [36]. In parallel, *PRG4* was upregulated in culture-expanded CCs, suggesting a progressive enrichment in chondroprogenitors. These findings are in line with the enhanced clonogenic ability and sustained expression of stemness markers in CCs throughout in vitro expansion, suggesting that this heterogeneous cell population conserves (or even expands) a chondrogenic progenitor pool of cells over time. It has been reported that normal CCs, OA CCs, and MSCs show no significant differences in CFU-F capacity when seeded in vitro directly after isolation [37]. Our data showed that CCs, ASCs, and BMSCs obtained from OA patients have comparable CFU-F capacity at P1, however, serial expansion of CCs resulted in CFU-F enrichment at P3. On the contrary, MSCs showed a tendency towards a loss of stemness potential during expansion, more dramatically observed in ASCs. Interestingly, *NANOG* and *POU5F1* expression became downregulated in cultured MSCs while it was maintained in CCs, thus reinforcing the concept of the presence of a pool of progenitor cells, such as CSPCs. 

Unlike with both MSCs and according to previous reports with sorted or clonal-selection obtained chondroprogenitors [8,9,11], our data demonstrated that CCs have little or no adipogenic potential. However, they were able to differentiate towards osteogenic and chondrogenic lineages, with the latter more pronounced than MSCs, as expected. 

The immunophenotyping analysis showed that OA CCs shared minimal MSC defining criteria for both negative (CD14, CD34, CD45) and positive (CD44, CD73, CD90, and CD105) markers [38], while positive for CD166^+^ in a higher proportion (28% ± 19%) than what was previously reported in normal AC (less than 10%) [8]. Another study showed that the cartilage of patients with severe end-stage OA is populated by a chondroprogenitor subpopulation (~32%) expressing CD9, CD90, and CD166 [9], data more comparable with our results overall. Moreover, in our CCs, we found a slightly higher proportion (~6%) of CD146, a marker that denotes a perivascular phenotype [17,39,40], in comparison with unsorted cells from diseased femoral condyles (0.5% and 2%) [41]. CD151, on the other hand, has been associated with undifferentiated states of BMSCs, which once subjected to chondrogenic differentiation, result in a significant reduction in its expression. Our results support the presence of undifferentiated chondroprogenitors as we found it high (~95%), similar to undifferentiated MSCs [42].

An exhaustive multiplex-based interrogation of the cell secretome before and after IL-1β stimulation allowed us to assess and compare the cell’s ability to respond to inflammatory environments. Under basal conditions, CCs exhibited the largest amount of secreted growth factors (GF) overall, with a special presence of chondrogenic, angiogenic, and pro-mitogenic molecules. This highly secretory profile may result from a long-standing exposure to a pathologic environment within the joint, inducing sustained reactive trophic secretory activity by CCs. This hypothetical scenario is supported by the fact that after ex vivo pro-inflammatory stimulation, no further increase in secretion of GFs was observed, suggesting that the cells already reached their secretory peak. Conversely, MSCs exhibited a significant boost (pronounced in ASCs and marginal in BMSCs) in their secretory response to IL-1β, doubling their secretion of GFs in the case of ASCs. That response includes trophic mediators for AC, such as factors belonging to IGF, PDGF, and TGFβ families, as well as angiogenic factors, reaching significant levels in CCs. This pro-angiogenic feature is likely related to the documented roles of MSCs in promoting tissue healing in general. However, considering the structural avascular nature of cartilage, this response might result in a metabolic alteration of tissue homeostasis. Along those lines, the high angiogenic factor secretion by CCs are likely to just be part of the overall highly secretory status of the cells, rather than a specific reaction to induce new vessels. Of note, angiogenesis in healthy cartilage is inhibited by anti-angiogenic mediators, while in inflammatory conditions, the increased production of angiogenic factors promotes pathological blood vessel formation [43]. 

A comparable induction of a migratory behavior may be related in all three cell types, as GM-CSF was massively upregulated after inflammatory stimulation. Furthermore, CCs and MSCs exposed to inflammatory conditions shared a catabolic behavior, with up-regulation of specific MMPs correlated with an enhanced migratory behavior of chondroprogenitor cells as previously reported; compared with “static” chondrocytes, migratory chondroprogenitors need to express metalloproteases 1, 3, 9, and 14 to penetrate through the ECM during migration [11].

The situation with the secretion of inflammation-modulating cytokines follows a trend in which all three cell types were reactive overall, with ASCs again more pronounced. Despite this comparable response, and contrary to the situation with GFs, CCs experienced increments in the secretion of a larger cohort of cytokines upon inflammatory stimulation. Multiple chemotactic and modulators of cellular components of innate and adaptive immune responses are modulated in all three cell types; however, the repertoire of molecules altered in CCs correspond more to an anti-inflammatory effect. Interestingly, IL-1Ra, a direct IL-1β antagonist, was significantly upregulated only in CCs and ASCs. 

The functional analysis of the secretome in the presence of activated PBLs evidenced a clear and significant abrogation of an activated T cell proliferative response by CCs. This unanticipated immunomodulatory effect exerted by CCs correlated with their induced secretome composition, and further provides evidence of functional similarities with MSCs. These findings have tremendous clinical implications as: First, the now evident intrinsic nature of AC to “fight” local immune responses, such as the ones present in inflammatory-mediated joint disease (e.g., rheumatoid arthritis) and early pathological events in OA [44,45], can become a target of exogenous interventions; second, the observed strong immunomodulatory capacity can also be harnessed during the design of cell-based therapy protocols to treat general AC damage; and third, to further extend their use to enhance tissue engineering-based protocols for focal AC injuries. In that respect, a popular yet suboptimal option is autologous chondrocyte implantation (ACI), which includes a heterogeneous CCs population that can be used in association with exogenous MSCs to induce a better phenotypic maintenance [46]. Along those lines, a previous study showed that infrapatellar fat pad-derived MSCs exposed to CCs extract from OA patients acquire a chondrocyte phenotype by upregulating major chondrogenic genes, such as *Sox-9, L-Sox5, Sox6*, and *Col2a1* [47]. 

A limitation of the present study is that only one in vitro model of inflammation was tested; nevertheless, IL-1β was chosen because it is the most used agent to induce in vitro inflammation to mimic OA [31,32]. Moreover, an in vivo functional test of the CCs is needed to better evaluate both their progenitor and therapeutic potentials. On the other hand, we acknowledge that CCs were taken from non-weight bearing areas of the femoral head, limiting the influence that the mechanical loading exerts on cellular responses.

## 5. Conclusions

In conclusion, our findings strengthen the hypothesis that CCs represent a tissue-specific target for future pharmacological and/or biophysical therapies. Moreover, given their particular features and performance, CCs from pathological joints may be an alternative therapeutic agent, which can be selectively isolated, enriched during expansion, and manufactured as a cell-based product or as its CM for AC damage cell therapy. However, the clinical utilization conditions and specifications will necessitate further studies, since the in vivo potential of the cells is still untested.

## Supplementary Materials

The following are available online at https://www.mdpi.com/2077-0383/8/4/423/s1, Figure S1: Growth factor secretion, Figure S2: Inflammation-related cytokines secretion, Table S1: List of proteins tested by growth factor array, Table S2: List of proteins tested by inflammatory array.
