# Supplementary material for: Human Diseased Articular Cartilage Contains a Mesenchymal Stem Cell-Like Population of Chondroprogenitors with Strong Immunomodulatory Responses
Source: J Clin Med. 2019 Mar 28;8(4):423. doi: 10.3390/jcm8040423 (PMC6517884; doi:10.3390/jcm8040423)
Supplement: Supplementary file 1 [file jcm-08-00423-s001.pdf]

Table S1. List of proteins tested by growth factor array.

Name	Function
**AR (Amphiregulin)**	Mitogenic
**EGF (Epidermal Growth Factor)**	Mitogenic, differentiation
**EGF-R (EGF Receptor)**	Binds EGF, mitogenic
**HB-EGF (heparin-binding EGF-like growth factor)**	Activates EGFR mediated signaling
**HGF (Hepatocyte Growth Factor)**	Mitogenic, angiogenesis, tissue regeneration
**NGFβ (beta Nerve Growth Factor)**	Mitogenic, differentiation
**FGFβ (beta Fibroblast Growth Factor)**	Mitogenic, angiogenic
**FGF-4**	Mitogenic, tissue regeneration
**FGF-6**	Mitogenic, tissue regeneration
**FGF-7/KGF (Keratinocytes Growth Factor)**	Mitogenic (keratinocytes), tissue regeneration
**G-CSF* (Granulocyte Colony Stimulating Factor)**	Granulocyte production, differentiation, function
**GM-CSF* (Granulocyte Macrophage Colony Stimulating Factor)**	Granulocyte and macrophage production, differentiation, function
**M-CSF* (Macrophage Colony Stimulating Factor)**	Macrophage production, differentiation, function
**M-CSF-R (Macrophage Colony Stimulating Factor Receptor)**	Binds M-CSF, macrophage production, differentiation, function
**IGFBP-1 (Insulin like Growth Factor Binding Protein 1)**	Binds IGF-1 and IGF-2, cell migration and metabolism
**IGFBP-2**	Binds IGF-1 and IGF-2
**IGFBP-3**	Binds IGF-1 and IGF-2, prolongs the half-life of IGFs and alters their interaction with receptors
**IGFBP-4**	Binds IGF-1 and IGF-2, prolongs the half-life of IGFs and alters their interaction with receptors

**IGFBP-6**	Binds IGF-2, association with the epithelial-mesenchymal transition
**IGF-1**	Growth and development
**IGF-sR (Insulin like Growth Factor 1 Receptor)**	Binds IGF-1
**IGF-2**	Growth and development
**NT-3 (neurotrophin 3)**	Survival and differentiation (neuron)
**NT-4**	Survival and differentiation (neuron)
**GDNF (Glial cell Derived Neurotrophic Factor)**	Survival, differentiation (neurons)
**PDGF-Rα (Platelet Derived Growth Factor Receptor alpha)**	Mitogenic (mesenchymal cells)
**PDGF-Rβ**	Mitogenic (mesenchymal cells)
**PDGF-AA (Platelet Derived Growth Factor subunit AA)**	Binds and activate PDGF receptor
**PDGF-AB**	Binds and activate PDGF receptor
**PDGF-BB***	Binds and activate PDGF receptor
**PLGF (Placental Growth Factor)**	Angiogenesis
**VEGF-A (Vascular Endothelial Growth Factor A)**	Angiogenesis
**VEGF-D**	Angiogenesis, lymphangiogenesis, endothelial cell growth
**VEGF-R2 (VEGF Receptor 2)**	Binds VEGFs
**VEGF-R3**	Binds VEGFs
**SCF (Stem Cell Factor, kit ligand)**	Pleiotropic
**SCF-R (SCF Receptor, CD117/c-kit)**	Binds SCFs
**TGFα (Transforming Growth Factor alpha)**	Mitogenic, differentiation
**TGFβ1***	Mitogenic, differentiation
**TGFβ2**	Mitogenic, differentiation
**TGFβ3**	Mitogenic, differentiation

*Evaluated also as anti-inflammatory proteins in inflammatory array.

Table S2. List of proteins tested by inflammatory array.

**Name**	**Function**
**Eotaxin**	Eosinophilic-mediated inflammatory diseases
**Eotaxin-2**	Chemotactic activity on resting T lymphocytes
**I-309**	Secreted by activated T cells, displays chemotactic activity for monocytes
**ICAM-1 (intercellular adhesion molecule 1)**	Expressed on endothelial cells and cells of the immune system
**IFN-γ (interferon gamma)**	Triggers a cellular response to viral and microbial infections
**1L-1α (interleukin 1 alpha)**	Produced by monocytes and macrophages
**IL-1β**	Produced by activated macrophages, mediator of the inflammatory response, involved in cell proliferation, differentiation, apoptosis
**IL-2**	Regulates proliferation of T and B lymphocytes
**IL-3**	Promotes cell growth
**IL-4**	Produced by activated T cells, overlapping functions with IL-13
**IL-6**	Produced at sites of acute and chronic inflammation, promotes the maturation of B cells
**IL-6-sR (interleukin 6 soluble receptor)**	Binds IL-6
**IL-7**	Regulates B and T cell development

**IL-8**	Secreted primarily by neutrophils, chemotactic factor, angiogenesis
**IL-10**	Immunoregulation
**IL-11**	Stimulates the T-cell-dependent development of immunoglobulin-producing B cells
**IL-12-p40**	Acts on T and natural killer cells, expressed by activated macrophages that serve as an essential inducer of Th1 cells development
**IL-12-p70**	Acts on T and natural killer cells, expressed by activated macrophages that serve as an essential inducer of Th1 cells development
**IL-13**	Produced primarily by activated Th2 cells, involved in B-cell maturation and differentiation, down-regulates macrophage activity, inhibits the production of pro-inflammatory cytokines and chemokines
**IL-15**	Regulates T and natural killer cell activation and proliferation
**IL-16**	Chemoattractant, modulator of T cell activation
**IL-17**	Proinflammatory cytokine produced by activated T cells, stimulates the expression of IL-6, cyclooxygenase-2, enhances the production of nitric oxide
**IP-10 (interferon-inducible protein 10)**	Stimulates monocytes, natural killer and T-cell migration, modulates the adhesion molecule expression
**MCP-1 (Monocyte Chemotactic Protein 1)**	Chemotactic activity for monocytes and basophils

**MCP-2**	Chemotactic activity for monocytes, lymphocytes, basophils and eosinophils
**MIG (Monokine-Induced by IFN-gamma)**	Chemoattractant for T cells
**MIP-1-α (Macrophage Inflammatory Proteins 1 alpha)**	Chemotactic, proinflammatory
**MIP-1-β**	Chemotactic, proinflammatory
**MIP-1-δ**	Chemotactic for T cells and monocytes
**RANTES (Regulated on Activation, Normal T Cell Expressed and Secreted)**	Chemoattractant for blood monocytes, memory T helpers, eosinophils
**TNF-α (Tumor Necrosis Factor alpha)**	Mainly secreted by macrophages, proinflammatory, regulates cell proliferation, differentiation, apoptosis, lipid metabolism
**TNF-β**	Produced by lymphocytes, mediates inflammatory, and immunostimulatory responses
**sTNF-RI (soluble Tumor Necrosis Factor Receptor I)**	Binds and inhibits TNF-a
**sTNF-RII**	Binds and inhibits TNF-a
**TIMP-2 (Tissue Inhibitor of Metalloproteinases 2)**	Inhibits the matrix metalloproteinases, suppress the proliferation of endothelial cells

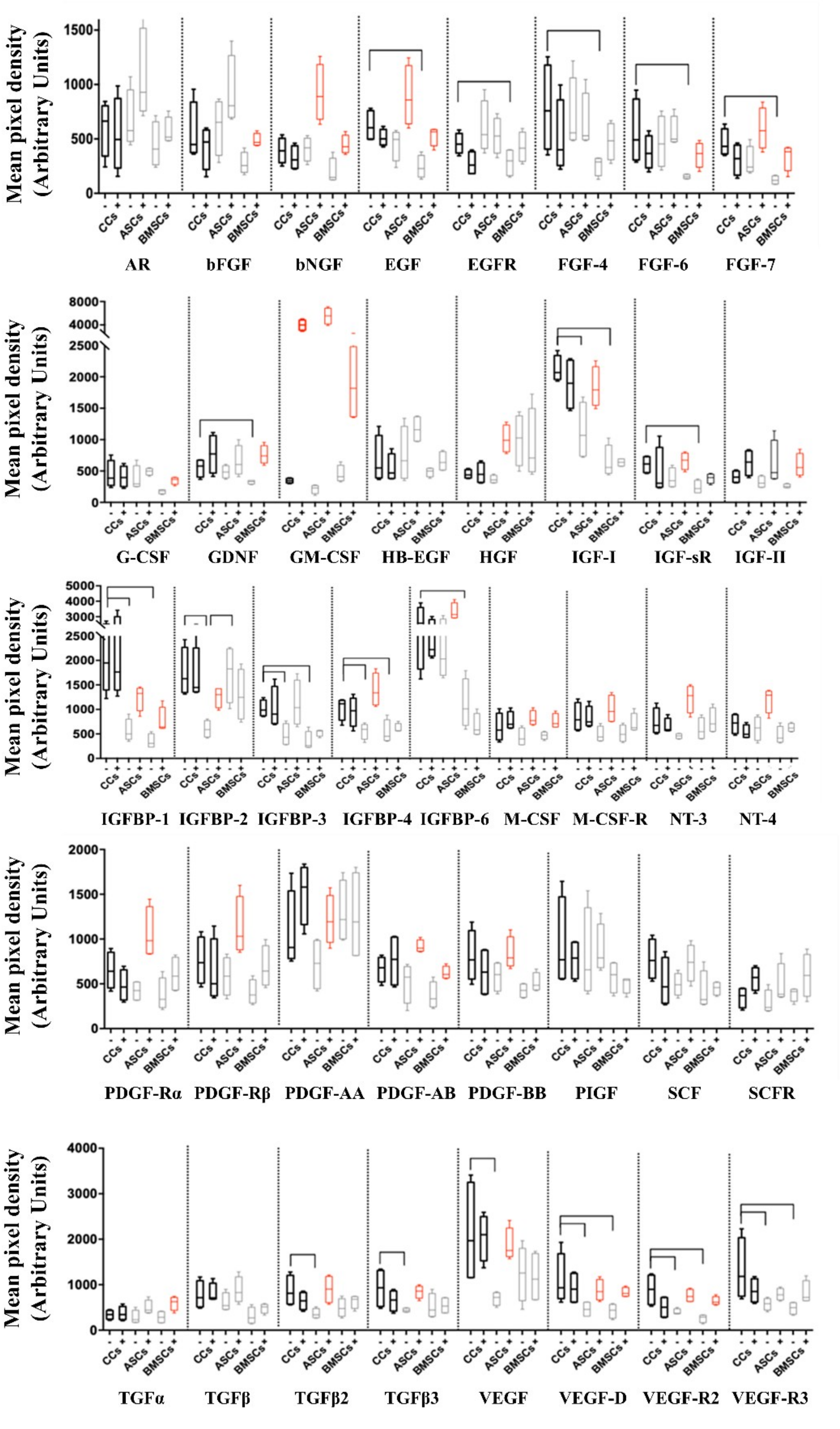

Supplementary Figure 1. Growth factors secretion. Adipose (ASCs)- and bone marrow (BMSCs)-derived MSCs and cartilage cells (CCs) at basal (-) and post-stimulation with IL-1β (+). Significant differences ($p<0.05$) in basal levels are evidenced by connecting lines, whereas differences between basal and post-stimulated samples are indicated as red boxes. Data are expressed as mean ± SD (n=4).

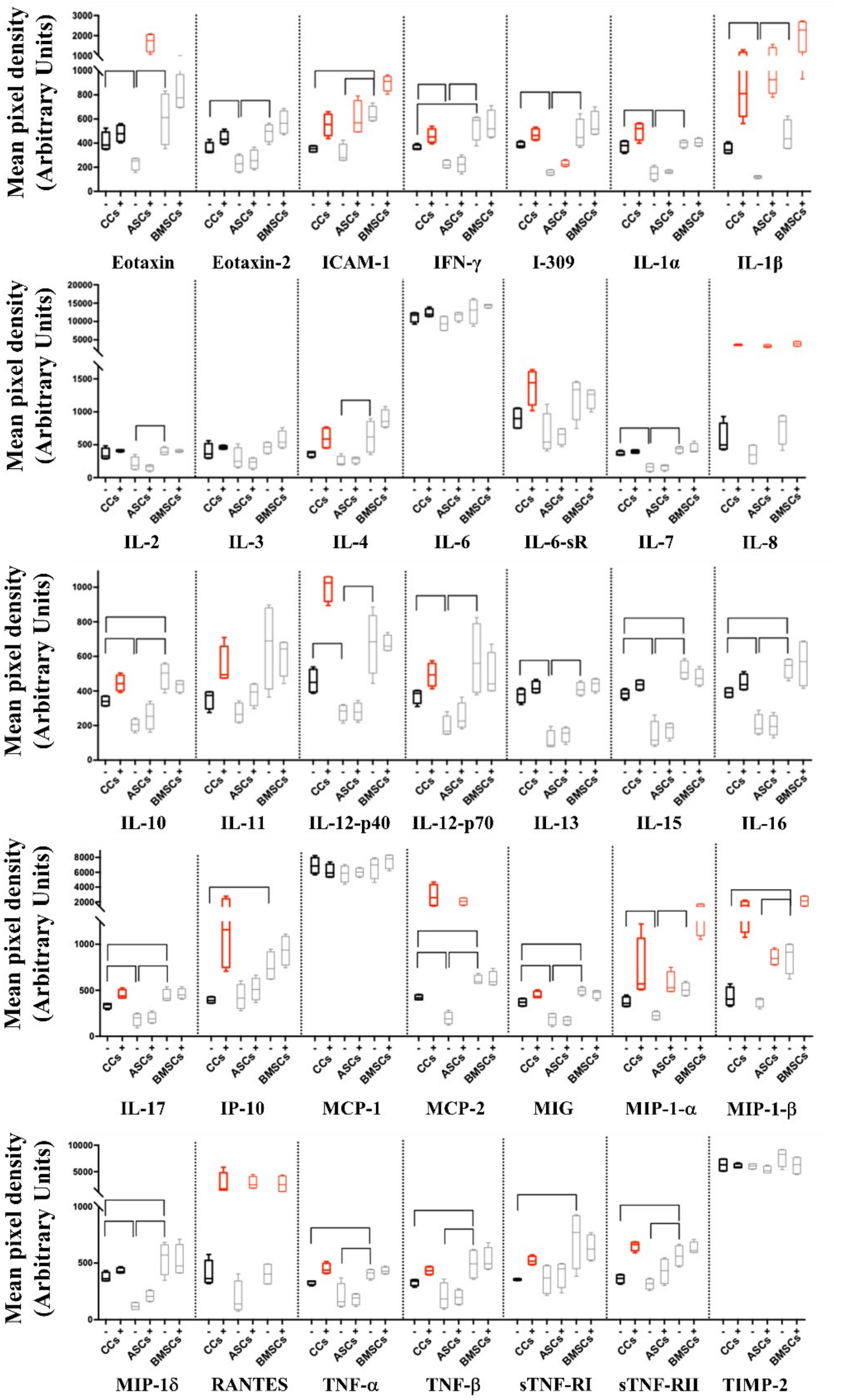

Supplementary Figure 2. Inflammation-related cytokines secretion. Adipose (ASCs)-and bone marrow (BMSCs)-derived MSCs and cartilage cells (CCs) at basal (-) and post-stimulation with IL-1β (+). Significant differences ($p<0.05$) in basal levels are evidenced by connecting lines, whereas differences between basal and post-stimulated samples are indicated as red boxes. Data are expressed as mean ± SD (n=4).
